# The first fungal laccase with an alkaline pH optimum obtained by directed evolution and its application in indigo dye decolorization

**DOI:** 10.1186/s13568-019-0878-2

**Published:** 2019-09-18

**Authors:** Qiang Yin, Gang Zhou, Can Peng, Yinliang Zhang, Ursula Kües, Juanjuan Liu, Yazhong Xiao, Zemin Fang

**Affiliations:** 1 School of Life Sciences, Anhui University, Hefei, 230601 China; 2 Anhui Key Laboratory of Modern Biomanufacturing, Hefei, 230601 China; 3 Institute of Physical Science and Information Technology, Anhui University, Hefei, 230601 China; 4 Molecular Wood Biotechnology and Technical Mycology, Büsgen-Institute, University of Goettingen, Büsgenweg 2, 37077 Göttingen, Germany; 5 Goettingen Center for Molecular Biosciences (GZMB), University of Goettingen, 37077 Göttingen, Germany

**Keywords:** Fungal laccase, Directed evolution, Alkaline pH activity, Indigo dye, Decolorization

## Abstract

Engineering of fungal laccases with optimum catalytic activity at alkaline pH has been a long-lasting challenge. In this study, a mutant library containing 3000 clones was obtained by error-prone PCR to adapt the optimum pH of a fungal laccase Lcc9 from the basidiomycete *Coprinopsis cinerea*. After three rounds of functional screening, a mutant with three amino acid changes (E116K, N229D, I393T) named PIE5 was selected. PIE5 showed an optimum pH of 8.5 and 8.0 against guaiacol and 2,6-DMP when expressed in *Pichia pastoris*, representing the first fungal laccase that possesses an optimum pH at an alkaline condition. Site directed mutagenesis disclosed that N229D contributed the most to the optimum pH increment. A single N229D mutation caused an increase in optimum pH by 1.5 units. When used in indigo dye decolorization, PIE5 efficiently decolorized 87.1 ± 1.1% and 90.9 ± 0.3% indigo dye at the optimum conditions of pH 7.0–7.5 and 60 °C, and with either methyl 3,5-dimethoxy-4-hydroxybenzoate or 2,2′-azino-bis(3-ethylbenzothazoline-6-sulfonate) as the mediator. In comparison, the commercially available fungal laccase TvLac from *Trametes villosa* decolorized 84.3 ± 1.8% of indigo dye under its optimum conditions (opt. pH 5.0 and 60 °C). The properties of an alkaline-dependent activity and the high indigo dye decolorization ability (1.3-fold better than the parental Lcc9) make the new fungal laccase PIE5 an alternative for specific industrial applications.

## Introduction

Laccases (benzenediol: oxygen oxidoreductases, EC 1.10.3.2) are a family of copper-containing oxidases (Baldrian 2006). They consist of three cupredoxin domain repeats D1 to D3 and contain four copper atoms as cofactors in two distinctive sites, with a type I copper center (T1) located in the core of D3, and a type II/type III trinuclear copper cluster (T2/T3) situated in a surface exposed cleft in between D1 and D3 (Hakulinen and Rouvinen 2015). Laccases can oxidize a variety of phenolic and non-phenolic substrates with or without mediators (Baldrian 2006). When oxidizing the substrate, electrons transfer from the substrate to the T1 Cu, then from the T1 site to the trinuclear copper cluster T2/T3, and eventually to a dioxygen molecule bound between the two T3 copper ions (Jones and Solomon 2015). Due to their strong oxidative power and with water as the final by-product, laccases are regarded as versatile enzymes for biotechnical applications such as pulp bleaching, textile refining, food upgrading, biofuel production, organic compound synthesis, bioremediation, and protective agents in cosmetics (Pezzella et al. 2015; Sitarz et al. 2016).

Laccases are widely distributed over bacteria, fungi, insects, and plant kingdoms (Hoegger et al. 2006; Kües and Rühl 2011). However, most of the laccases characterized until now are of bacterial or fungal origin due to their ease of production and purification (Baldrian 2006; Chauhan et al. 2017). Typically, bacterial laccases have a low-redox potential (340–470 mV), while their fungal counterparts have middle- (490–710 mV) and high-redox potentials (730–790 mV) (Rodgers et al. 2010; Mate and Alcalde 2015). The higher redox-potential and specific activity, and a broader substrate spectrum of fungal laccases make them much more attractive in terms of specific industrial applications than the corresponding bacterial laccases (Rodgers et al. 2010; Pezzella et al. 2015).

One of the most intriguing characteristics of fungal laccase catalysis is its pH dependence. Most fungal laccases display toward phenolic substrates an optimum activity in the pH range of 3.0–5.5 and they become essentially inactive as the pH is approaching to neutral and alkaline, although they are structurally stable above 7.0 (Xu 1997; Baldrian 2006). On the other hand, fungal laccases with high activity at neutral/alkaline pH are highly desirable, especially for their applications in specific industrial processes (Madzak et al. 2006; Torres-Salas et al. 2013; Novoa et al. 2019). For example, during a bioethanol production process using steam-exploded wheat straw as the substrate, treatment with laccase at alkaline condition (pH 8) resulted in a reduction in lignin content in the solid fraction and in an increase in both glucose and xylose production after a saccharification step (De La Torre et al. 2017). Alkaline-tolerant (pH 9) laccase is more preferred in hair coloring industries (Endo et al. 2012; Fang et al. 2014). Furthermore, fungal laccases with high activity at blood pH (pH 7.4) also show potential to contribute to the technology revolution of implantable self-contained wireless 3D nano-biodevices that work in different physiological fluid (Mate et al. 2013a). At the present time, the main technological applications of fungal laccases are still in the textile industries, for example in eco-friendly denim destaining (Pezzella et al. 2015; Muñoz et al. 2017). Experience showed that degradation of indigo dye by alkaline active fungal laccases (pH > 7) results in less background staining of jeans as compared with the practice conducted in acidic conditions. Destaining at neutral pH could thus provide products with better added-values (Colomera and Kuilderd 2015). Unfortunately, no effective fungal laccases with optimum pH at neutral/alkaline conditions have been reported from nature and could so far neither be created in the laboratory despite of much efforts of mutagenesis (Maté et al. 2010, 2013a, b; Mate and Alcalde 2015; Novoa et al. 2019).

Previously, we obtained a laccase named Lcc9 from the basidiomycete *Coprinopsis cinerea* (Pan et al. 2014) which is naturally expressed by the fungus (in the further called wLcc9 for wild-type Lcc9) upon induction by *p*-hydroxybenzoic acid (HBA) (Hu et al. 2019). Lcc9 recombinantly produced in *Pichia pastoris* (rLcc9) showed an optimum pH of 6.5 towards phenolic substrates such as guaiacol after heterologous expression in *P. pastoris* (Xu et al. 2019). In this study, error-prone PCR and screens in *P. pastoris* were used for directed protein mutagenesis to increase the laccase activity of Lcc9 in alkaline pH. One positive mutant enzyme (designated as PIE5) with an optimum pH of 8.5 towards guaiacol was obtained. The application potential of PIE5 on indigo dye decolorization was also evaluated. PIE5 represents the first fungal laccase with an alkaline pH optimum and can serve as candidate for specific applications at alkaline conditions.

## Materials and methods

### Strains, chemicals, and culture media

*Escherichia coli* JM109, *P. pastoris* GS115 and the plasmid pPIC9K were purchased from Invitrogen (Carlsbad, CA, USA). Guaiacol, 2,6-dimethoxyphenol (2,6-DMP), and 2,2′-azino-bis(3-ethylbenzothazoline-6-sulfonate) (ABTS) were obtained from Sigma-Aldrich (St. Louis, MO, USA). The error-prone PCR kit was acquired from Aviva Systems Biology (Beijing, China). All other chemicals and reagents were of analytical grade. Yeast Extract Peptone Dextrose Medium (YPD), Minimal Dextrose Medium (MD), Buffered Glycerol-Complex Medium (BMGY) and Buffered Minimal Methanol Medium (BMM) were prepared following the instructions from Invitrogen.

### Mutation library construction and screening

Error-prone PCR based mutagenesis was conducted using a cloned *lcc9* cDNA sequence as the template and a primer pair of *lcc9*F and *lcc9*R as primers (Xu et al. 2019) (Additional file 1: Table S1). The final MnCl_2_ concentrations in the amplification system were 0.6, 0.7, 0.8, 0.9, and 1.0 mM, respectively. The mutated cDNA fragments amplified by the PCR were recovered, mixed together, digested with *Not*I and *Eco*RI, and ligated into the pPIC9K vector subjected to the same digestion treatment, generating a library of plasmids pPIC9K-m*lcc9* with mutated *lcc9* cDNA fragments. The pPIC9K-m*lcc9* library was transformed into competent *E. coli* JM109 cells and screened using Amp. All Amp-resistant *E. coli* JM109 clones were harvested and plasmids were extracted, which were then linearized by using *Sac*I and transformed into *P. pastoris* GS115 by electroporation. The electroporated cells were plated onto MD agar plates for selecting the His^+^ transformants. All transformants were selected and grown on BMGY plates at 28 °C for 2 days and kept at − 80 °C as a mutation library for future usage. *P. pastoris* GS115 transformed with the empty vector pPIC9K (*P. pastoris* GS115/pPIC9K) and pPIC9K-*lcc9* with the original *lcc9* cDNA (*P. pastoris* GS115/pPIC9K-*lcc9*) were used as negative and positive controls in the following screenings.

For the first-round screening, the strains were cultivated for 4 days at 28 °C on BMMC agar plates containing 0.2 mM ABTS. Secretion of active laccase, with methanol induction, was identified by the presence of a dark green zone around transformant colonies. Positive clones were selected and in second-round screening cultivated for 48 h at 28 °C in shaken 96-well plates (200 rpm) containing 50 μL BMGY medium in each well. Then, 150 μL liquid BMM medium was added into each well to induce laccase expression. After incubating at 28 °C and 200 rpm for another 48 h, the plates were centrifuged at 2500×*g* for 5 min to obtain the supernatants, which were transferred into new plates for laccase activity determinations. The assay system for the second-round screening contained citrate/phosphate buffer (pH 6.5 or 8.5) and 10 mM guaiacol in a total volume of 200 μL added to each well. The absorbance was monitored at 465 nm and 30 °C for 10 min. Mutants with higher activity at pH 8.5 than at pH 6.5, and with higher activity at pH 8.5 than the positive control *P. pastoris* GS115/pPIC9K-*lcc9* were selected for further screening. In the third-round screening, positive colonies were cultured in shaking flasks, enzyme expression was methanol-induced and mutant proteins were purified to homogeneity as described in Xu et al. (2019) for optimum pH determination.

### Site-directed mutagenesis

Specific mutants of Lcc9 were constructed based on site-directed mutagenesis using respective primers (Additional file 1: Table S1) and the plasmid pPIC9K-*lcc*9 as the template (Xu et al. 2019). Positive *P. pastoris* strains harboring the intended mutant genes were obtained as described above and verified by sequencing.

### Laccase expression and purification

Laccase expression in *P. pastoris* was conducted according to Xu et al. (2019). The wild-type Lcc9 (wLcc9; GenBank: DAA04514.1) expressed by *C. cinerea* was obtained by cocultivation *C. cinerea* Okayama 7 with *Gongronella* sp. w5 and purified according to the method of Pan et al. (2014).

When purifying rLcc9 and mutant laccases, the aqueous culture broth was centrifuged at 8000×*g* for 10 min. Culture supernatants were collected and concentrated to 100 mL in a Minitan Ultrafiltration System with a regenerated low-binding cellulose membrane (Millipore, Bedford, MA, USA). The concentrate was centrifuged at 12,000×*g* for 20 min, and the supernatant was then dialyzed against citrate/phosphate buffer (20 mM, pH 7.5) overnight, followed by centrifugation again at 12,000×*g* for 20 min. Then, the supernatant was applied to a DEAE-Sepharose FF column (10 × 200 mm, Amersham Pharmacia, Uppsala, Sweden) and eluted according to protocols reported previously (Xu et al. 2019).

The homogeneity of the purified protein was determined by sodium dodecyl sulfate (SDS) polyacrylamide gel electrophoresis (PAGE) with a 12% polyacrylamide gel and stained with Coomassie Brilliant Blue R-250. The protein concentration was assayed using the Bradford method, with bovine serum albumin as standard (Sangon Biotech, Shanghai, China). Native PAGE was conducted using guaiacol as the substrate as described previously (Pan et al. 2014). The purified laccase was identified according to Rühl et al. (2013) by LC–ESI–MS/MS (LTQ, Thermo Fisher Scientific, Shanghai, China). Proteins were then identified by searching the data against a database of the *C. cinerea* Okayama 7 (#130) (Stajich et al. 2010).

### Enzyme assay

The assay mixture consisted of 10 μL of appropriately diluted culture supernatant or enzyme stock and 990 μL of 100 mM citrate/phosphate buffer (pH 4.0) containing 5 mM guaiacol. It was measured at 465 nm for 5 min at 30 °C (*ε*_465_ = 12,000 M^−1^ cm^−1^) (Froehner and Eriksson 1975). Alternative substrates for the measurement of laccase activity were 0.5 mM ABTS (*ε*_420_ = 36,000 M^−1^ cm^−1^) (Paice and Bourbonnais 1990) and 10 mM 2,6-DMP (*ε*_468_ = 49,600 M^−1^ cm^−1^) (Fang et al. 2015). Heat-treated laccase was used as the control. One activity unit (U) was defined as the amount of laccase protein required for oxidizing 1 μmol of substrate per minute. All experiments were performed in triplicate.

### Characterization of mutant laccases

The effect of pH on laccase activity was assayed in 50 mM citrate/phosphate buffer (4.0–8.0) and 50 mM Tris/HCl buffer (8.0–9.5) at 60 °C. The effect of temperature was determined by incubating protein in the temperature range of 4–80 °C at the optimum pH for each substrate. The enzyme stabilities against pH and temperature were determined by incubating proteins at various temperatures and different pH values, and then the residual activities were determined as mentioned above. All experiments were performed in triplicate.

The kinetics and the specific activities toward guaiacol and 2,6-DMP of rLcc9 and the seven distinct mutants created in this study were measured at the optimum pH and temperature of each mutant. The kinetic constants (*K*_m_, *k*_cat_, and *k*_cat_/*K*_m_) were determined by incubating proteins with various concentrations of guaiacol and 2,6-DMP.

### Redox-potential determination

The redox-potential of laccases was measured using cyclic voltammetry at pH 6.5. The pyrolytic graphite electrode (PGE, GaossUnion, Shanghai, China), platinum wire electrode (ChenHua, Shanghai, China), and calomel electrode (ChenHua) were used as the working electrode, counter electrode, and reference electrode, respectively. Before testing, the PGE was prepared by ultrasonic cleaning for 5 min with 95% ethanol and H_2_O, respectively. Then, 5 μg laccase was dripped on the PGE and placed at 4 °C until the laccase was completely adsorbed on the PGE. Then, the redox potentials of the samples were detected using an electrochemical workstation (CHI660D, Chenhua, China).

### Sequence alignment and protein structure modeling

The most similar protein to PIE5 was a laccase (Lcc4) from *Lentinus* sp. that has 56% sequence identity (PDB id:3X1B) to *C. cinerea* wLcc9 (GenBank: DAA04514.1) with its crystal structure resolved at 1.8 Å (Maestre-Reyna et al. 2015). A PIE5 model was thus generated by using the automated Swiss-Model protein modeling server and *Lentinus* Lcc4 as the template (http://swissmodel.expasy.org/) and analyzed with the PyMol software.

### Indigo dye decolorization

The mutant PIE5, wLcc9, and rLcc9 were used for indigo dye (Sigma-Aldrich) decolorization. Initially, the test system contained citrate/phosphate buffer (50 mM, pH 7.0), enzyme (200 mU mL^−1^), and indigo dye (200 μM) in a total volume of 1 mL. The reaction mixtures were incubated at 40 °C (except for temperature optimization reactions) for 2 h. Mediators (final concentration, 100 μM) including 3,5-dimethoxy-4-hydroxybenzaldehyde (DHB), syringic acid (SA), methyl 3,5-dimethoxy-4-hydroxybenzoate (MS), 1-hydroxybenzotriazole (HBT), and ABTS were used to evaluate the effect of mediator on decolorization. The effects of pH (4.5–9.0), temperature (20–80 °C), mediator concentration (20–300 μM), reaction time (0–180 min), and enzyme concentration on dye decolorization (40–200 mU mL^−1^) were evaluated one by one based on a single factor optimization strategy. All assays were carried out in triplicate. The decolorization rates of indigo dye by laccases were monitored according to the decrease in absorbance at 665 nm. The heat-treated enzymes were used as the negative controls.

Reactions using the commercial fungal laccase from *Trametes villosa* (Tlac, provided by Novozymes, Tianjin, China) were used as the positive controls. Briefly, when 200 μM ABTS was used as the mediator, the test system contained 200 mU mL^−1^ Tlac and 200 μM indigo dye in a total volume of 1 mL. Reaction was conducted at pH 7.0 and 60 °C for 180 min. When 120 μM MS was used as the mediator, reaction was conducted at pH 7.5 and 60 °C for 50 min. Meanwhile, reaction was also conducted at pH 5.0 and 60 °C for 3 h according to the manufacturer’s instruction, without addition of any mediator.

The decolorization ratio was calculated according to the following equation

$${\text{Decolorization ratio (\%)}} = ({{\text{A}}} - {{\text{A}}_{0}} )/{{\text{A}}}_{{0}} \times 100\%$$

A_0_ and A represent the initial and final absorbance of indigo dye, respectively.

## Results

### Laccase mutation library construction and positive clone screen

Lcc9 cDNA was previously successfully expressed in *P. pastoris* (Xu et al. 2019). It was used as the template for directed evolution to improve the pH optimum of Lcc9 to alkaline condition. Based on the error-prone PCR technique, a Lcc9 cDNA mutation library was obtained which contained 3000 *P. pastoris* colonies with an average mutation rate of 4.63 nucleic acids per gene copy, according to the sequencing of 60 randomly picked colonies (data not shown).

**Fig. 1:**
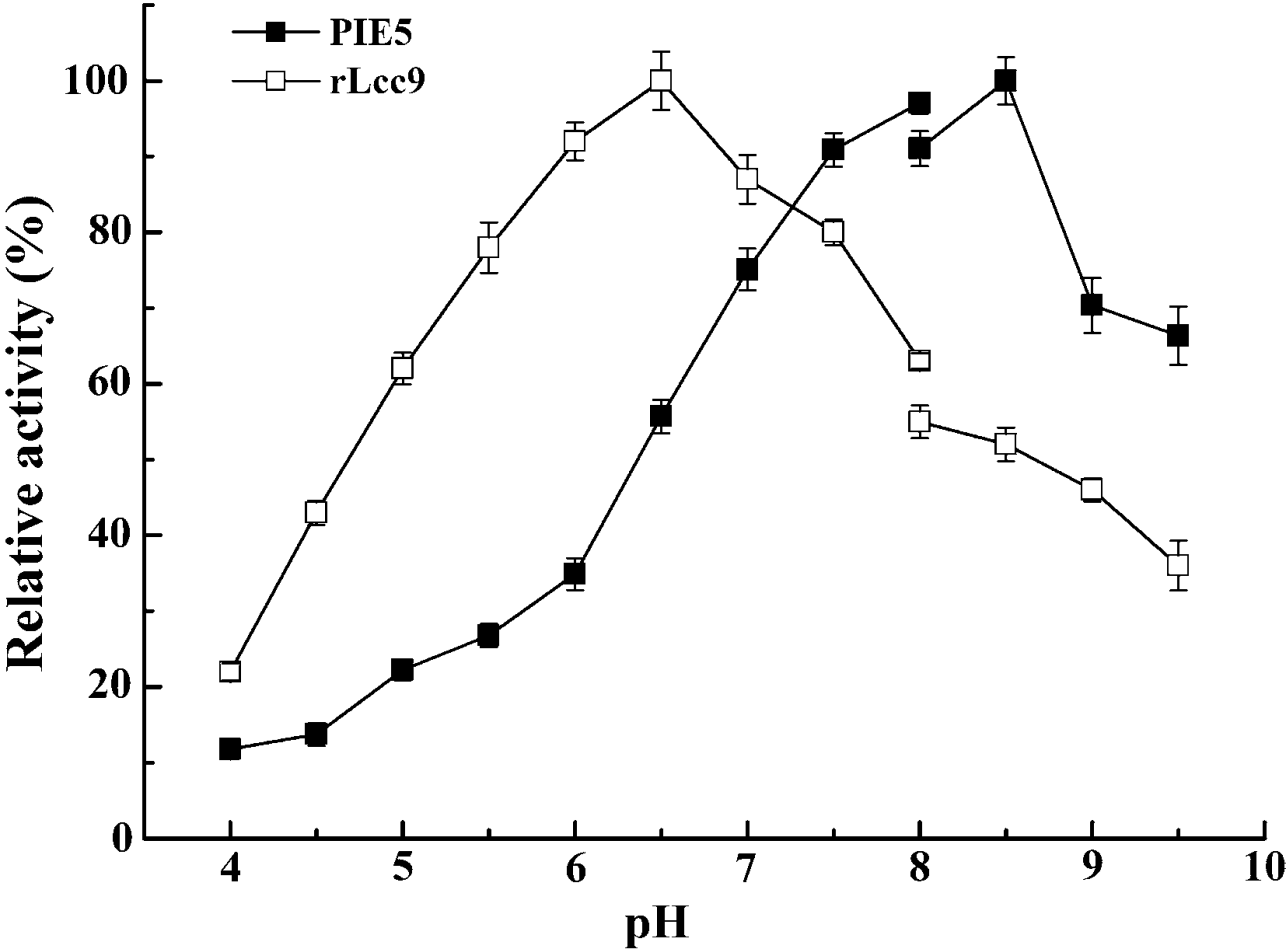
The pH profiles of PIE5 and rLcc9 towards guaiacol. The data presented are the average values from triplicate parallel experiments and technical repeats of measurements (n = 9)

Phenotype analysis was used to select the target mutants in the first-round functional screening. A total of 1960 colonies, which showed a dark-green halo around the colony after 4 days cultivation on the BMMC agar plates of stronger intensity than the control with plasmid pPIC9k were recognized as positive colonies and picked up for future screening. In the second-round multi-well plate screening, four positive colonies with at least 10% higher laccase activity at pH 8.5 than at 6.5 and at least 10% higher activity at pH 8.5 than the control rLcc9 colony were screened out based on activity determination using guaiacol as the substrate. In the third-round liquid fermentation screening, one mutant protein, named PIE5, with an optimum pH of 8.5 was obtained. PIE5 retained more than 70% of original activity in the pH range of 7.0–9.5 (Fig. 1). In comparison, rLcc9 showed an optimum pH of 6.5 and retained approximately 60% of the original activity in the pH range of 5.0–8.0 (Fig. 1 and Xu et al. 2019). In the slightly acidic pH range at pH 5.0 to pH 6.5, enzyme activities measured for PIE5 were lower, between ca 20 and 55% of optimum activity.

### Biochemical property comparison between PIE5 and rLcc9

**Fig. 2:**
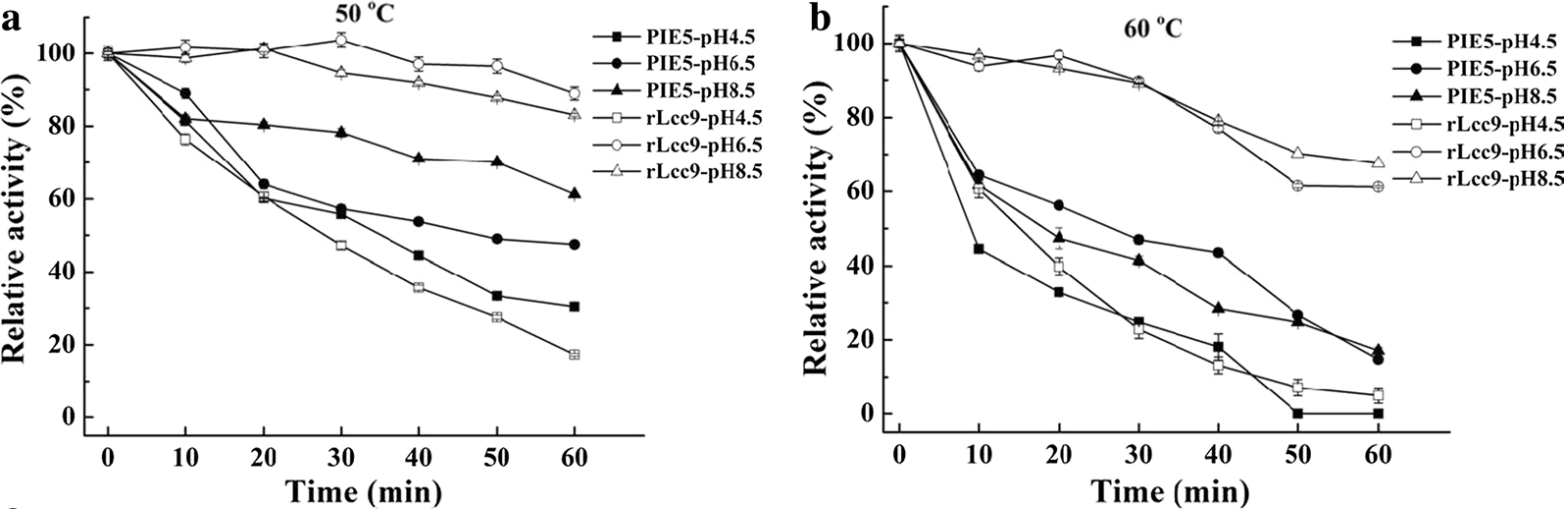
pH stabilities and thermostabilities of rLcc9 and PIE5 as determined at incubation temperatures of 50 °C (**a**) and 60 °C (**b**), respectively. The data presented are the average values from triplicate parallel experiments and technical repeats of measurements (n = 9)

PIE5 showed an optimum catalytic temperature at 60 °C (Table 1), which was the same to that of rLcc9 (Xu et al. 2019). However, the temperature and pH stabilities of the PIE5 were quite different from those of rLcc9. Generally, rLcc9 was more stable than PIE5 both at pH 6.5 and 8.5 and at temperatures of 50 °C and 60 °C, respectively (Fig. 2). At 50 °C, PIE5 was more stable at pH 8.5 than that at 4.5 and 6.5, with 65% of the original activity that retained after 1 h incubation at pH 8.5. PIE5 showed a half-life time of 20 min and 30 min, respectively, after incubation at 60 °C and pH 8.5 and 6.5. In comparison, rLcc9 retained more than 85% of activity after 1 h incubation at 50 °C and pH 6.5 and 8.5, and it retained more than 60% of activity after 1 h incubation at 60 °C and pH 6.5 and 8.5 (Fig. 2). These results suggested that PIE5 showed a strong reduction in both its thermostability and pH stability as compared to rLcc9.

**Table 1:** The optimum pH, temperature, redox potential, and kinetic constants of rLcc9, PIE5, and its mutants on two substrates

Proteins	Specific activity (U mg^−1^)^a^	Redox potential (mV)^b^	Guaiacol					2,6-DMP				
			pH	Temp. (°C)	*K*_m_ (M)	*k*_cat_ (S^−1^)	*k*_cat_/*K*_m_ (M^−1^ S^−1^)	pH	Temp. (°C)	*K*_m_ (M)	*k*_cat_ (S^−1^)	*k*_cat_/*K*_m_ (M^−1^ S^−1^)
rLcc9	315.3	505.7	6.5	70	0.9 × 10^−4^	22.8	2.5 × 10^5^	6.5	60	2.3 × 10^−3^	69.4	3.0 × 10^4^
PIE5	318.4	598.9	8.5	60	3.3 × 10^−4^	62.9	1.8 × 10^5^	8.0	60	5.7 × 10^−3^	43.5	7.5 × 10^3^
E116 K	251.2	528.3	6.5	60	3.2 × 10^−4^	85.8	2.5 × 10^5^	7.0	60	1.6 × 10^−3^	75.2	4.5 × 10^4^
N229D	477.9	575.4	8.0	60	3.7 × 10^−4^	87.3	2.3 × 10^5^	8.0	60	2.6 × 10^−3^	66.2	2.5 × 10^4^
I393T	173.8	550.2	7.0	60	1.4 × 10^−4^	83.9	5.7 × 10^5^	7.0	60	1.2 × 10^−3^	49.2	3.8 × 10^4^
E116K/N229D	253.2	582.7	8.0	60	3.4 × 10^−4^	75.4	2.1 × 10^5^	8.0	60	3.0 × 10^−3^	38.0	1.2 × 10^4^
E116K/I393T	176.5	515.7	6.5	60	1.1 × 10^−4^	46.0	3.9 × 10^5^	7.0	60	1.5 × 10^−3^	34.1	2.1 × 10^4^
N229D/I393T	363.3	556.5	8.0	60	3.4 × 10^−4^	82.5	2.3 × 10^5^	8.0	60	2.9 × 10^−3^	38.8	1.3 × 10^4^

The data presented are the average values from triplicate parallel experiments and technical repeats of measurements (n = 9)

^a^Determined using ABTS as the substrate

^b^Determined at pH 6.5

The specific activity of PIE5 was 318.4 U mg^−1^ towards ABTS, comparable to that of rLcc9 (315.3 U mg^−1^, Xu et al. 2019). The kinetic properties of PIE5 and rLcc9 were determined under optimum conditions (Table 1). When using guaiacol and 2,6-DMP as the substrates, the *K*_m_ of PIE5 increased 3.7-fold (3.3 × 10^−4^ M) and 2.5-fold (5.7 × 10^−3^ M), compared to those of rLcc9, which were 0.9 × 10^−4^ and 2.3 × 10^−3^ M, respectively. PIE5 showed a 2.8-fold increase in *k*_cat_ on guaiacol compared to that of rLcc9. This result translated to a similar catalytic efficiency of PIE5 compared to that of rLcc9. In comparison, when using 2,6-DMP as a substrate, PIE5 showed a 1.6-fold decrease in *k*_cat_, resulting in a fourfold decrease in catalytic efficiency as compared to that of rLcc9 (Table 1). The better performance of PIE5 against guaiacol than against 2,6-DMP may be attributed to the fact that guaiacol was used as the selection substrate throughout the screening process (Madzak et al. 2006).

### Sequence analysis of PIE5

PIE5 contained three mutations in the sequence, including E116K, N229D, and I393T. Sequence alignment was conducted among *C. cinerea* Lcc9 and other fungal laccases with known structures presented in the PDB database. Inspection of the sequence alignments of the laccase family revealed that the position 116 is located between the two laccase signature regions L1 and L2 for copper-binding (Kües and Rühl 2011) including the consensus motifs VN(T)QCPI and WYHSH. Furthermore, position 116 is quite variable in laccase sequences: L, S, or D are the main residues that occur at this position. In comparison, E is present in Lcc9 at this position (Additional file 1: Fig. S1). Differently from the loop region with E116 in between L1 and L2, the positions 229 and 393 are highly conserved among laccases from basidiomycetes. For example, position 393 in Lcc9 is occupied by an I, which is not found in many other laccases at the same position (Additional file 1: Fig. S1) while e.g. in some putative laccases from related species of the *Psathyrellaceae* family (not further shown). As an alternative, a V is often encoded at this position in other fungal laccase genes of other families of *Basidiomycetes* (Additional file 1: Fig. S1).

**Fig. 3:**
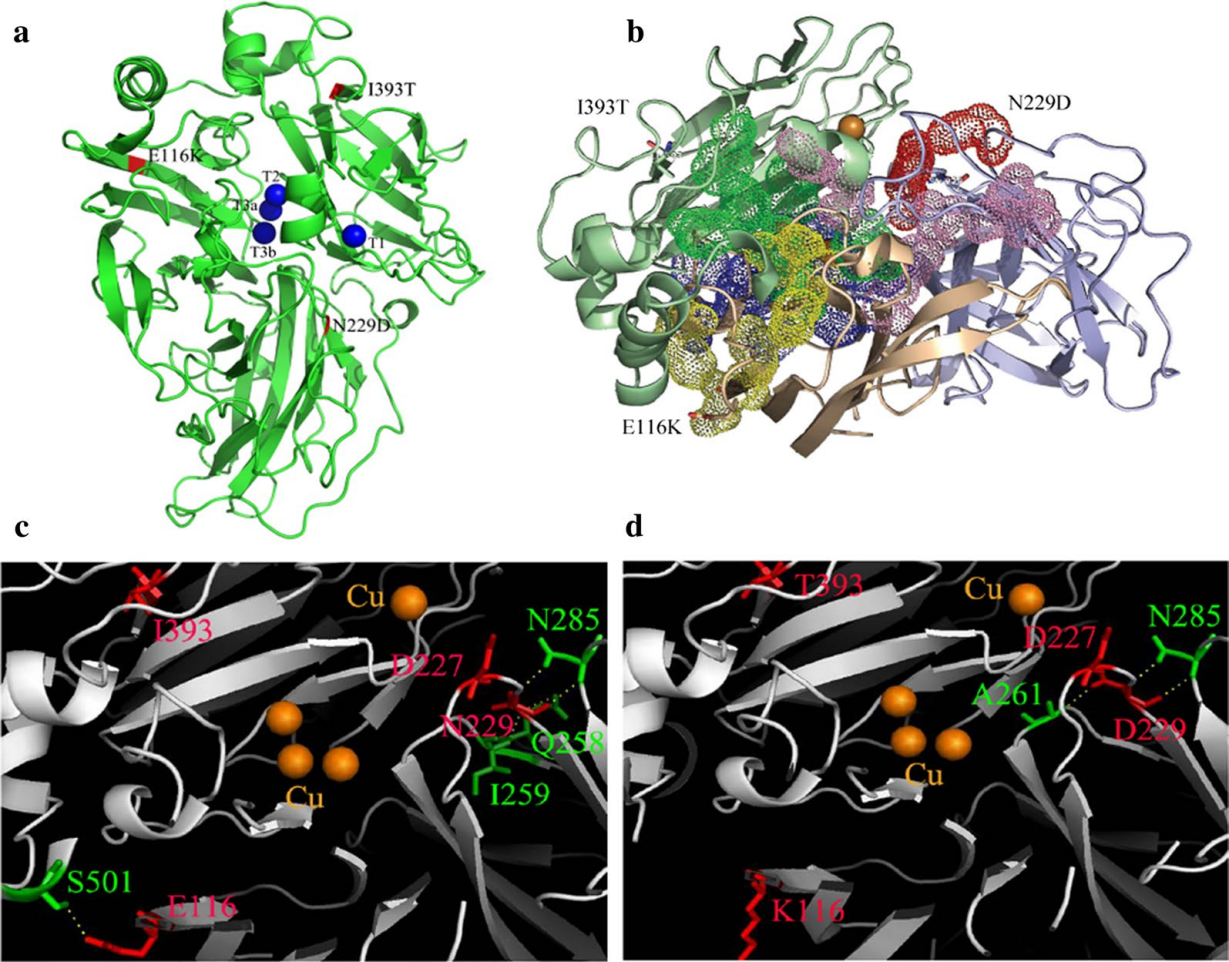
Structural simulation of PIE5. **a** Ribbon representation of PIE5 3D structure model with the mutated residues. **b** Small molecule transport channels in PIE5. Green: channel p1; Red: channel p2; Pink: channel p3; Blue: channel p4; Yellow: channel p5. **c**, **d** Stereo views of the local sites in *C. cinerea* rLcc9 (**c**) and the mutated sites in variant PIE5 (**d**)

Homology modeling of the PIE5 structure was employed using the laccase from *Lentinus* sp. (PDB ID: 3X1B, 56% sequence identity to the wild-type Lcc9) as a template to further understand the contribution of the three potentially beneficial mutations in PIE5 (Maestre-Reyna et al. 2015). The three mutated sites were distributed over different domains of the protein. Accordingly, E116K and I393T were both located on the surface of the protein, and were far from the substrate binding area, the copper centers, and the water channel, respectively (Fig. 3a). However, the negatively charged E116 on the laccase domain D1 of the wildtype Lcc9 enzyme was H-bonded with the polar S501 from the laccase domain D3, suggesting that they may contribute to a pH-dependent 3-dimensional stability of the protein by the formation of interdomain bridges (Herrera-Zúñiga et al. 2019). After the substitution to the positively charged K, the H-bond was abolished (Fig. 3c, d and Additional file 1: Fig. S2).

I393T was located on the first loop region that connects the β-sheet on D3 (Fig. 3). No convincing structural or interaction change was found for I393T with the change from the hydrophobic I to the polar T (Fig. 3 and Additional file 1: Fig. S2). However, five transportation channels (P1–P5) were recently predicted based on the random acceleration molecular dynamic simulations (Li and Zhao 2018). Based on the PIE5 model, I393T was located close to the oxygen entrance of channel P1 (Fig. 3b), which is recognized as the oxygen channel with the highest efficiency (Piontek et al. 2002). As a result, we deduced that I393T may affect the oxygen transfer from the outside to the T3 Cu^2+^.

N229D was found in a random coil of the notch inside of the protein in D2 (Fig. 3a). Position 229 may participate in the formation of the optimal binding pocket for a specific substrate (Pardo et al. 2016; Mehra et al. 2018). In the wildtype Lcc9, N229 interacted with the surrounding residues Q258, I259 and N285 through a complex network of H-bonds (Fig. 3c, d). After mutation, except for N285, the H-bonds of D229 with the surrounding residues Q258 and I259 were abolished. Meanwhile, D229 formed a new H-bond with A261, which was located near the T2/T3 copper center (Fig. 3c, d). It seems that a new linkage was made as follows: D229-A261-G262-W129-H87-T2/T3 copper center, which means that the mutation N229D may indirectly affect the T2/T3 copper center (Additional file 1: Fig. S2).

### Characterization of the three PIE5 mutations in single and double mutants

Six different mutants, namely E116K, N229D, I393T, E116K/N229D, E116K/I393T, and N229D/I393T, were constructed based on site-directed mutations. They were expressed in *P. pastoris*, purified, and biochemically characterized following the protocols as described in “Materials and methods” section (Table 1 and Additional file 1: Fig. S3).

Biochemical characterization showed that the six mutated proteins showed different optimum pHs. Among them, E116K and E116K/I393T showed an optimum pH of 6.5 toward guaiacol, similar to that of rLcc9, indicating that position E116K had little impact on increasing the optimum pH to alkaline. N229D contributed the most to the optimum pH increment, because both the single-point mutation N229D and the two-point mutations including N229D each caused a 1.5 units increment in the optimum pH in activity toward guaiacol. I393T showed an optimum pH of 7.0 towards guaiacol. E116K, I393T, and E116K/I393T showed an optimum pH of 7.0 toward 2,6-DMP, compared to 6.5 of rLcc9. In comparison, mutants including N229D, E116K/N229D, and N229D/I393T shared the same optimum pH of 8.0 with that of PIE5 (Table 1). All the six mutants shared the same optimum temperature of 60 °C with rLcc9 and PIE5 (Table 1).

As measured with ABTS, the specific activities were 251.2, 477.9, 173.8, 253.2, 176.5, and 363.3 U mg^−1^ for E116K, N229D, I393T, E116K/N229D, E116K/I393T, and N229D/I393T, respectively. Under optimum conditions, the kinetic constants of the six mutated proteins were tested and listed in Table 1. I393T and E116K/I393T showed a 2.5 to 3-fold increase in substrate affinity when compared to PIE5, more similar to rLcc9, whereas other mutations showed no apparent changes in substrate affinity toward guaiacol as compared to PIE5. Except for E116K/I393T, which showed a *k*_cat_ of 46 s^−1^, other mutants showed *k*_cat_ values of 75.4 to 87.3 s^−1^, higher than that of PIE5, which was 62.9 s^−1^. It should be noted that the catalytic efficiencies of I393T and E116K/I393T were 5.7 × 10^5^ and 3.9 × 10^5^ M^−1^ s^−1^, three- and twofold higher than that of PIE5, respectively.

### Redox potentials of the proteins

The redox potentials of the proteins mentioned above were tested using a cyclic voltammetry method at pH 6.5. PIE5 showed a redox potential of 598.9 mV, much higher than that of rLcc9, which was 505.7 mV under the same test condition (Table 1). The redox potential of the six mutated proteins were ranged from 515.7 to 582.7 mV, and were lower than that of PIE5, but higher than that of rLcc9 (Table 1).

### Indigo dye degradation

**Fig. 4:**
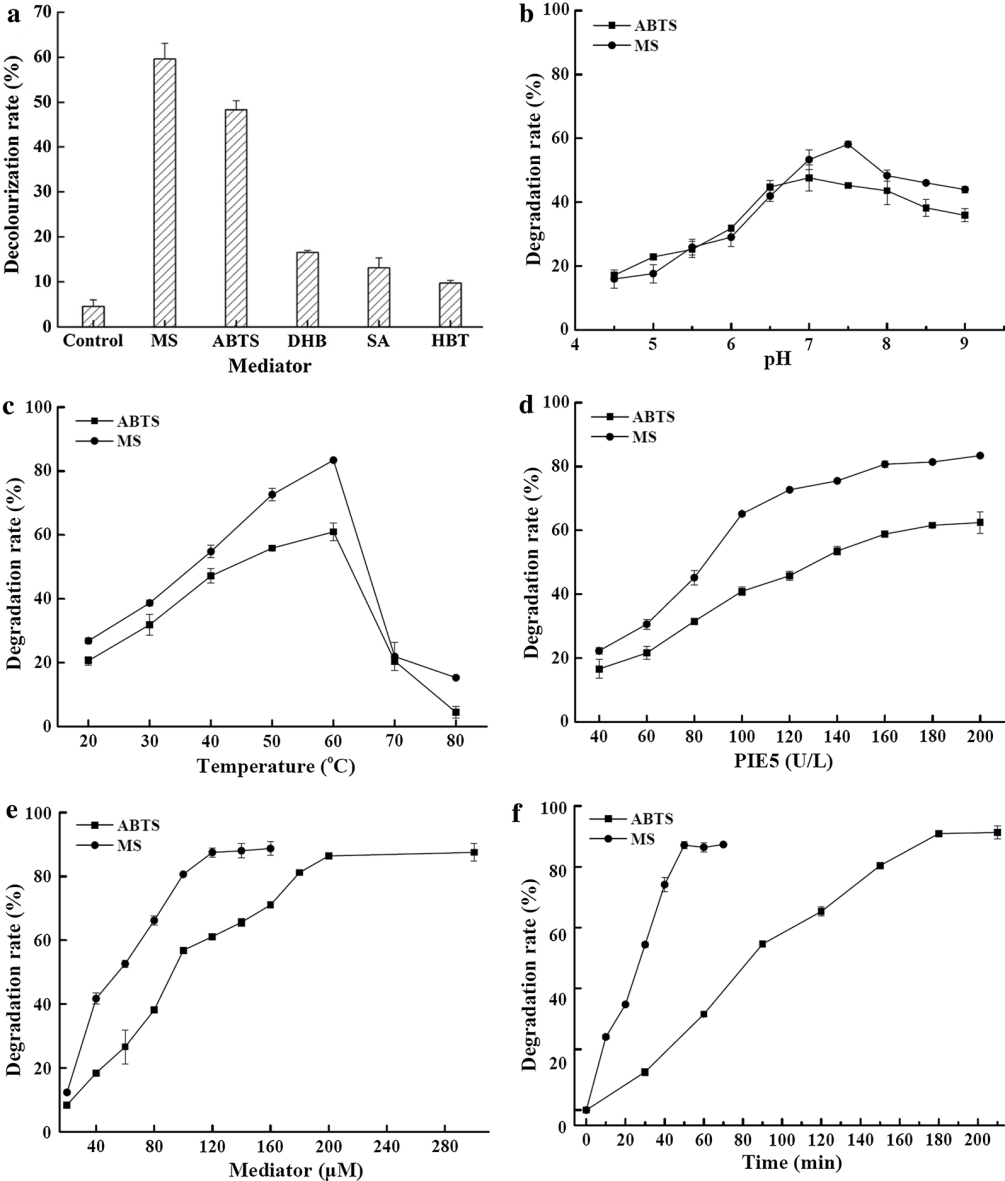
Indigo dye decolorization condition optimization by PIE5. **a** Mediator type, **b** pH, **c** temperature, **d** PIE5 dosage, **e** mediator dosage, **f** incubation time. The data presented are the average values from triplicate technical repeats of measurements (n = 9)

PIE5 hardly oxidized indigo dye in the absence of mediator, with only 5% indigo dye that was decolorized after 2 h incubation under initial test conditions (Fig. 4a). Addition of mediators including MS, DHB, SA, HBT, and ABTS promoted the decolorization rates of PIE5 on the indigo dye. MS was the best mediator for decolorization under the initial test conditions. The decolorization rate for PIE5 in the presence of MS was 59.7 ± 3.5%. Using ABTS as the mediator, the decolorization rate was 48.4 ± 2.0%. Mediators to improve indigo dye decolorization by PIE5 were ranked as follows: MS > ABTS > DHB > SA > HBT (Fig. 4a).

The conditions for PIE5 to decolorize indigo dye were further optimized by using a single factor optimization method. PIE5 showed a high decolorization rate at neutral/alkaline conditions. When using MS and ABTS as the mediators, the optimum pH and temperature for PIE5 to decolorize indigo dye were pH 7.5 and 60 °C, and pH 7.0 and 60 °C, respectively (Fig. 4b, c). PIE5 decolorized about 40% of 200 μM indigo dye even at pH 9.0 after incubation at 40 °C for 2 h (Fig. 4b). The performance of PIE5 was positively associated with the mediator dosage (Fig. 4d), PIE5 concentration (Fig. 4e), and incubation time (Fig. 4f). Finally, in the presence of 120 μM MS, 200 mU mL^−1^ PIE5 decolorized 87.1 ± 1.1% of 200 μM indigo dye after 50 min of incubation at pH 7.5 and 60 °C (Fig. 4f). When using 200 μM ABTS as a mediator, 200 mU mL^−1^ PIE5 decolorized 90.9 ± 0.3% of 200 μM indigo dye after incubating at pH 7.0 and 60 °C for 3 h (Fig. 4f), which was much better than the performances of wLcc9 and rLcc9, which were 70–72% and 75–79%, respectively, under the same conditions (Table 2).

**Table 2:** Comparison of the decolorization rates of indigo dye with four types of laccases

	Mediator (μM)	Opt. pH	Time (min)	Decolorization rate (%)				
				wLcc9	rLcc9	PIE5	Tlac	Tlac^a^
ABTS	200	7.0	180	71.9 ± 2.2	75.8 ± 0.2	90.9 ± 0.3	69.6 ± 1.3	84.3 ± 1.8
MS	120	7.5	50	70.2 ± 0.7	78.8 ± 0.9	87.1 ± 1.1	59.3 ± 1.8	

The data presented are the average values from triplicate parallel experiments and technical repeats of measurements (n = 9)

^a^Reaction was conducted at pH 5.0 and 60 °C according to the manufacturer’s instruction, using 200 μM indigo and 200 U L^−1^ enzyme for 3 h, without addition of any mediator

The performance of PIE5 on indigo dye decolorization was further compared to that of commercial *T. villosa* laccase from Novozymes. Under the conditions suggested by the producer at pH 5.0, 200 mU mL^−1^ *T. villosa* laccase decolorized 84.3 ± 1.8% of 200 μM indigo dye after incubation at 60 °C for 3 h. Furthermore, the *T. villosa* laccase decolorized 69.6 ± 1.3% and 59.3 ± 1.8% of 200 μM indigo dye, respectively, under the same conditions as optimized for PIE5 (Table 2).

## Discussion

Fungal laccases have been extensively studied over the past decades and recognized as being one of the ‘greenest’ catalysts (Pezzella et al. 2015). However, one of the major obstacles that prevented rapid progress of fungal laccase in applications in modern industries is their acidic pH dependence (Madzak et al. 2006; Torres-Salas et al. 2013; Novoa et al. 2019). This is because most of the fungal laccases show an optimum pH of 3–5.5 over phenolic substrates and they rapidly lose their activities at pH > 7 (Xu 1997; Xu et al. 1998). In consequence, fungal laccases with optimum pH and high activity at neutral or alkaline conditions are highly desirable, especially for applications in specific industrial processes (Madzak et al. 2006; Torres-Salas et al. 2013; Schwaneberg et al. 2018).

Several studies have reported the improvement of the optimum pH of fungal laccases. For example, mutation of basidiomycetous laccase OB-1 resulted in the change of the optimum pH from 4.0 to 5.0–6.0 in reactions with 2,6-DMP as substrate. At pH 7.0, the mutated protein retained 50% of its original activity for 2,6-DMP, whereas the wild-type protein displayed negligible activity under these conditions (Maté et al. 2010). However, all the engineered fungal laccases still have an optimum pH at acidic pHs (Cusano et al. 2009; Mate et al. 2013a, b; Torres-Salas et al. 2013; Novoa et al. 2019), even though one mutant named L365E/L513M of laccase MaL-M1 from *Melanocarpus albomyces* showed a threefold improved *k*_cat_ compared to the wild type laccase at pH 9.8 (Novoa et al. 2019). In this study, PIE5, a mutant fungal laccase from *C. cinerea* with remarkably improved enzymatic activity at alkaline pH, was obtained based on error-prone PCR and high-throughput functional screening. PIE5 showed an optimum pH of 8.5 toward guaiacol, and retained more than 70% of its activity at pH range of 7–9.5 (Fig. 1). Thus, PIE5 is regarded as an efficient catalyst at alkaline conditions. To the best of our knowledge, PIE5 is the first fungal laccase that has an optimum alkaline pH toward guaiacol. The optimum pH of 8.5 is even higher than those of certain bacterial laccases which are well known to have optimum pHs at neutral or slightly alkaline conditions, for example Ibh1 from *Bacillus halodurans* (optimum pH 7.5–8) (Ruijssenaars and Hartmans 2004), Ppo1 from *Marinomonas mediterranea* (around pH 7) (Jimenez-Juarez et al. 2005), and Lac15 from a marine metagenome (pH 7.5) (Fang et al. 2011).

According to Xu (1997), both the redox potential difference [ΔE_0_ = E_0_ (laccase T1) − E_0_ (substrate, single electron)] between a reducing substrate and the T1 center, and the OH^−^ inhibition at the T2/T3 center affect the pH activity profile of a laccase towards phenolic substrates: the former positively correlates with the reaction rate and mainly affects the ascending part of the pH profile curve. On the contrary, when the pH increases, the T2/T3 cluster can accept hydroxyl ions, which competes with the ΔE_0_ contribution, interrupts the trafficking of electrons from T1 Cu, and decreases laccase activity. In this study, both the rLcc9 and PIE5 showed bell-shaped pH profiles toward guaiacol (Fig. 1), indicating that the two physicochemical factors both affect the PIE5 pH profile. From our data, all the mutation proteins showed higher redox potentials than rLcc9 (Table 1), and these connected with the observed optimum pH values of each protein, revealing that the redox potential may be responsible for the optimum pH increment. This may be explained by using Xu’s conclusions: if the redox-potential increases for a laccase, the phenomenon that the enzyme activity decreases during a pH increment may be overcome (Xu 1997; Torres-Salas et al. 2013). However, it should be noted that complex mechanisms other than redox potential also play important roles in the pH effects on fungal laccases against phenol substrates, as indicated in other reports. For example, Cusano et al. (2009) succeeded in shifting the pH profile of a laccase toward an alkaline condition, with which the redox potential of the laccase decreased. In comparison, laccase variant ChU-B exhibits a higher activity at neutral/alkaline pH values than the wild-type laccase from basidiomycete PM1, while keeping the same redox potential (Mate et al. 2013b).

Our results showed that PIE5 contained 3 mutations in the amino acid sequence compared to Lcc9. Based on the biochemical characterization of both single and double mutation proteins, position N229D played the major impact on the pH profile of PIE5 (Table 1). However, mutations at this specific side in laccase have so far not been mentioned in the literature. Position N229 is highly conserved among basidiomycete laccases and located in a conserved random coil of the notch that participates in substrate binding and contributes to the substrate electron transfer (Fig. 3 and Additional file 1: Fig. S2) (Bertrand et al. 2002; Mehra et al. 2018). After the amino acid mutation of N229 to D229, the calculated pI of its side chain changed from 6.3 to 8.6. Usually, amino acids should be freely switched between protonation/deprotonation in a corresponding pH range to facilitate the electron transfer from the substrate to the T1Cu (Shigeno et al. 2015; Magni et al. 2018). In consequence, we suggest that the change from N to D may affect the association/dissociation of amino acids surrounding D229 at pH 8.5, and then substrate recognition guiding and the subsequent substrate electron transmission, as it is also suggested from the altered kinetic values of mutants carrying D229 (Table 1). Indeed, replacing the D with N at position 227 of laccase TvLB from *Trametes versicolor* changed the optimum pH of the enzyme against 2,6-DMP from pH 3.4 to 4.8, because of favoring substrate deprotonation (Madzak et al. 2006). Moreover, it was reported that the general increase in activity of the variant was due to a conformational rearrangement of the T2/T3 copper coordination sphere (Xu 1997; Mate et al. 2013a; Torres-Salas et al. 2013; Scheiblbrandner et al. 2017). Protein structure assimilation results showed that N229 in wild-type *C. cinerea* Lcc9 is in complicated manner connected with surrounding residues by using H-bonds (Additional file 1: Fig. S2). The network was changed after the D229 mutation, with some H-bonds abolished and one newly formed. These changes may indirectly affect the microenvironment of the T2/T3 copper center, the election transfer, and OH^−^ binding at the T2/T3 center, and finally this will affect the PIE5 pH profile against substrates such as guaiacol and 2,6-DMP (Table 1).

Other than the N229D mutation, also the single I393T mutation but not the E116K mutation caused a change in the optimum pH on activity against guaiacol by 0.5 units (Table 1). This suggests that also the change I393T will contribute to the change in the optimum pH in PIE5. I393 was found located on the surface of the Lcc9, at long distances to the substrate binding area, the copper centers, and the water channel. No convincing structural changes were found for I393T. However, I393 was found located near the best oxygen transfer channel (Fig. 3b). Given that the dependence of O_2_ reduction potential on pH could also impact the pH activity profile (Xu 1997), we speculate that the I393T mutation may cause an allosteric effect of PIE5 and affected the transfer of oxygen to the trinuclear copper center, and finally affected the electron transfer from substrate to T1 copper center, T2/T3 center, and to oxygen. As a support to this hypothesis, we found that redox potential of I393T increased to 550.2 mV. The substrate affinities of the three mutants containing I393T also increased 2.5 to 3-folds (Table 1).

Laccases are proteins with highly conserved and functionally essential regions. In consequence, not many positions can be mutated without changing activities (Bloom and Arnold 2009). From structural simulation data, E116 may contribute to formation of interdomain bridges between D1 and D3 (Herrera-Zúñiga et al. 2019). As a result, the identified serendipitous mutation E116K, although not directly involved in catalysis, may be involved in influencing the pH profile of the enzyme by influencing protein structure and stabilization (Maté et al. 2010, 2013a; Scheiblbrandner et al. 2017).

At present, main technological applications of fungal laccases are in the textile industry (Pezzella et al. 2015). They are used in the enzymatic degradation of indigo dye in both the stone-wash process and the deinking treatment of dyeing effluents (Mishra et al. 2011). In fact, after the first commercial product based on fungal laccase was launched in 1996 by Novozymes, several types of fungal laccases have been used in textile industries (Rodriguez-Couto 2012). However, most commercially used fungal laccases have a very narrow optimum pH range of 3.5–5.5 toward phenolic substrates, such as guaiacol (http://www.brenda-enzymes.org/), and they lose their activities at neutral or alkaline pH (Xu 1997), thus limiting their application in modern industries (Madzak et al. 2006). Our results showed that PIE5 can decolorize highly efficiently indigo dye under neutral and alkaline conditions (Fig. 4). Compared to other fungal laccases, PIE5 showed a higher decolorization rate, while the decolorization required less mediator and enzyme (Campos et al. 2001). Furthermore, our results also showed that PIE5 possessed a similar or higher indigo dye decolorization rate (working at pH 7.0–7.5) compared to the commercial *T. villosa* laccase from Novozymes (working at optimum pH 5.0) (Table 2). Fungal laccases with high activity and stability at neutral and alkaline pHs are highly desirable for textile industries, because the commonly used acidic reaction conditions of fungal laccases in textile industries for deinking and bleaching require subsequent neutralization steps after application, which in turn results in high amounts of salts and leads to disposal and pollution problems. As increasing environmental considerations are concerned, eco-friendly processes are attracting more and more public attention. In addition, in textile staining, indigo dye usually shows better solvation at neutral and alkaline pH and causes less background staining of the jeans compared with the practice conducted in acidic conditions. Deinking by laccases at alkaline or neutral pH provides therefore products with higher added-values. Decolorization of indigo dye at neutral pH means a reduction in the use of neutralization chemicals and also less back-staining of the textiles by the indigo dye (Colomera and Kuilderd 2015). In conclusion, our results proved that the newly developed laccase variant PIE5 with an alkaline optimum pH and a better decolorization percentage for indigo dye has a good potential in such types of industrial applications.

## Supplementary information

**Additional file 1: Table S1.** Primers used in this study. **Fig. S1.** Sequence alignment of Lcc9 and laccase sequences from other basidiomycetes. ▲ indicates the mutated amino acids. **Fig. S2.** Stereo views of the three types of cupper binding sites in *C. cinerea* Lcc9 and the mutated sites in variant PIE5. The copper atoms, one type-1 (T1), one type-2 (T2), and, two type-3 (T3a and T3b), are coordinated by the surrounding ten conserved histidines, one conserved cysteine and two water molecules. Protein residues are shown as a link model, the oxygen atoms are shown in red, nitrogen in blue, sulfur in yellow, and carbon in green. The four copper ions and water (Wat1 and Wat2) molecules are represented by blue and red spheres, respectively. a, rLcc9; b, PIE5. **Fig. S3.** Native PAGE analysis of the purified rLcc9, PIE5, and the specific mutants. Proteins were stained with 1 mM guaiacol in citrate/phosphate buffer (pH 4.0).

## Data Availability

Not applicable.
