# Supplementary material for: The first fungal laccase with an alkaline pH optimum obtained by directed evolution and its application in indigo dye decolorization
Source: AMB Express. 2019 Sep 18;9:151. doi: 10.1186/s13568-019-0878-2 (PMC6751238; doi:10.1186/s13568-019-0878-2)

**Additional file**

**AMB Express**

The first fungal laccase with an alkaline pH optimum obtained by directed evolution and its application in indigo dye decolorization

Qiang Yin^a,b^, Gang Zhou^a,b^, Can Peng^a,b^, Yinliang Zhang^a,b^, Ursula Kües^d,e^, Juanjuan Liu^a,b^, Yazhong Xiao^a,b*^, Zemin Fang^a,b,c*^

a, School of Life Sciences, Anhui University;

b, Anhui Key Laboratory of Modern Biomanufacturing;

c, Institute of Physical Science and Information Technology, Anhui University, Hefei, Anhui 230601, China

d, Molecular Wood Biotechnology and Technical Mycology, Büsgen-Institute, University of Goettingen, Büsgenweg 2, 37077, Goettingen, Germany;

e, Goettingen Center for Molecular Biosciences (GZMB), University of Goettingen, 37077, Goettingen, Germany

*, Correspondence

Phone/Fax: +86 551 63861063

E-mail: zemin_fang@ahu.edu.cn (to FZ); yzxiao@ahu.edu.cn (to XY)

Table S1 Primers used in this study

| Primer | Sequence (5’----3’) |
| --- | --- |
| *lcc9*F | CGGAATTCCAAATCCTTGGCCCG |
| *lcc9*R | AAGGAAAAAAGCGGCCGCTTAAGGAGTGG |
| 116-F | GCATTGAACCTGTACTTAAACGACTCTC |
| 116-R | GAGAGTCGTTTAAGTACAGGTTCAATGC |
| 229-F | GAACTTGTAGTCGGGATCGCACG |
| 229-R | TCGTGCGATCCCGACTACAAGTTC |
| 393-F | GGCAAAGTATGGGTCGCACCAC |
| 393-R | AGTGGTGCGACCCATACTTTGCC |

Fig. S1 Sequence alignment of Lcc9 and laccase sequences from other basidiomycetes. ▲ indicates the mutated amino acids.


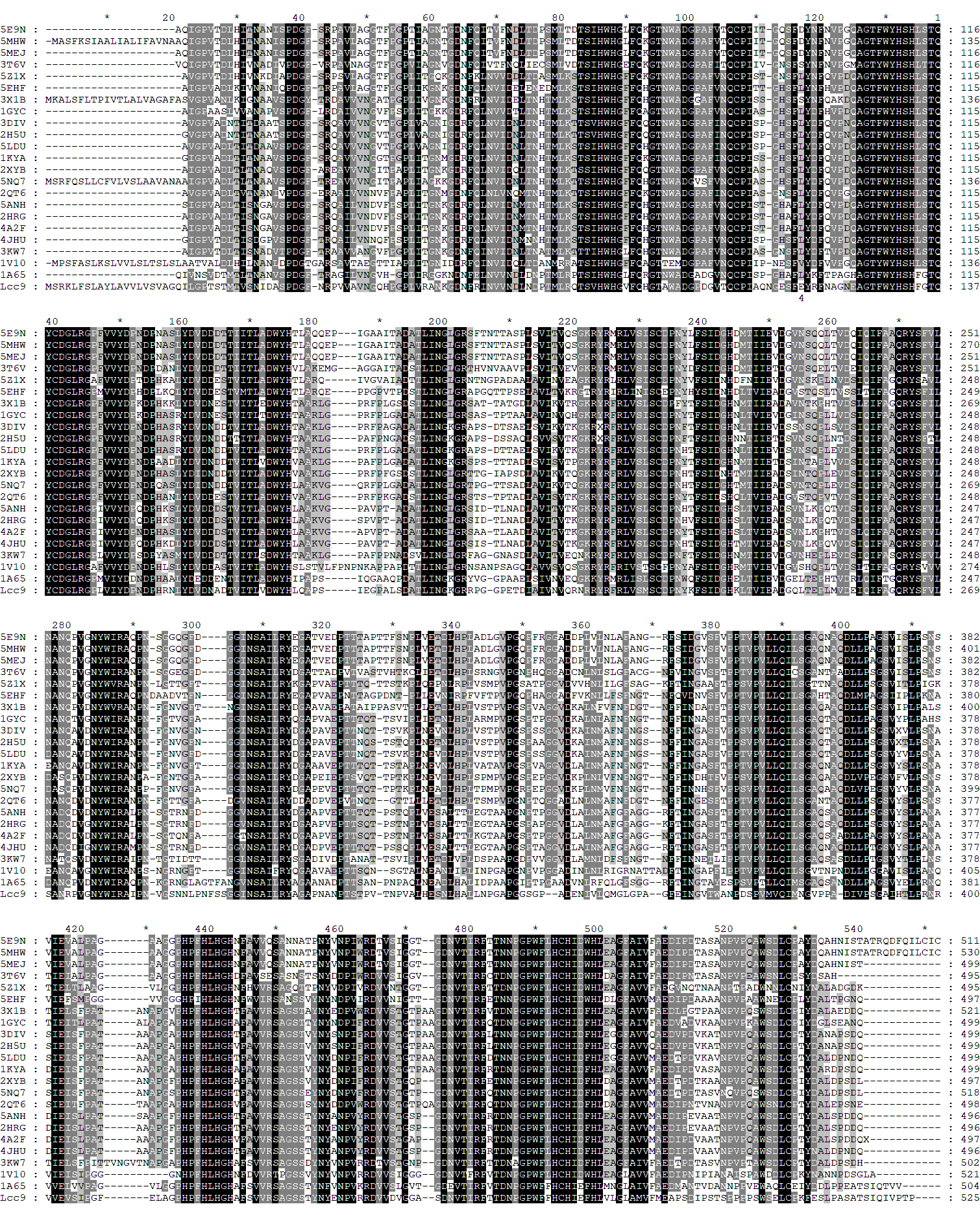


Fig. S2 Stereo views of the three types of cupper binding sites in *C. cinerea* Lcc9 and the mutated sites in variant PIE5. The copper atoms, one type-1 (T1), one type-2 (T2), and, two type-3 (T3a and T3b), are coordinated by the surrounding ten conserved histidines, one conserved cysteine and two water molecules. Protein residues are shown as a link model, the oxygen atoms are shown in red, nitrogen in blue, sulfur in yellow, and carbon in green. The four copper ions and water (Wat1 and Wat2) molecules are represented by blue and red spheres, respectively. a, rLcc9; b, PIE5.


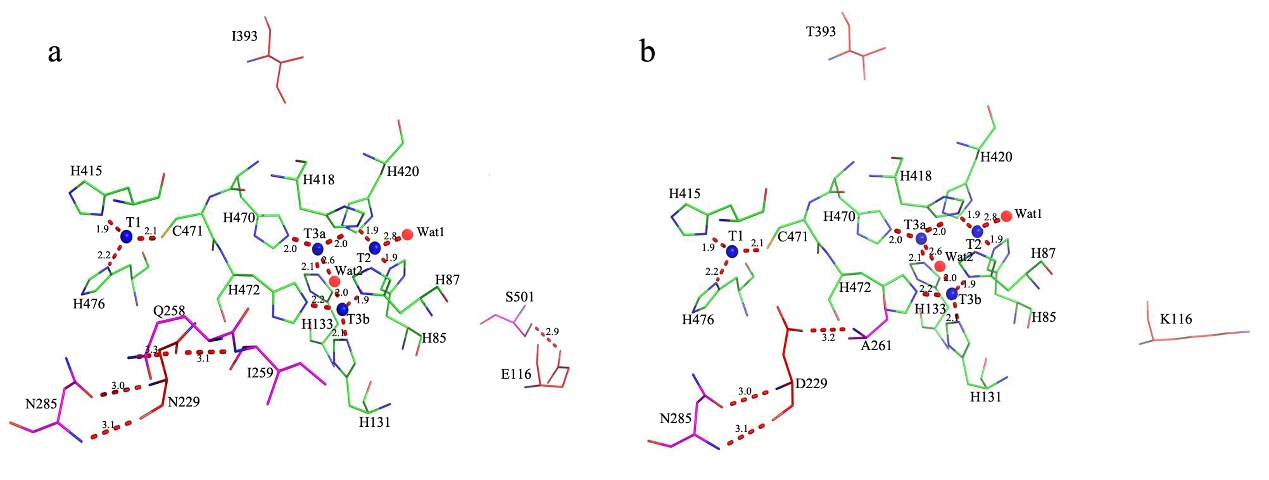


Fig. S3 Native PAGE analysis of the purified rLcc9, PIE5, and the specific mutants. Proteins were stained with 1 mM guaiacol in citrate/phosphate buffer (pH 4.0).


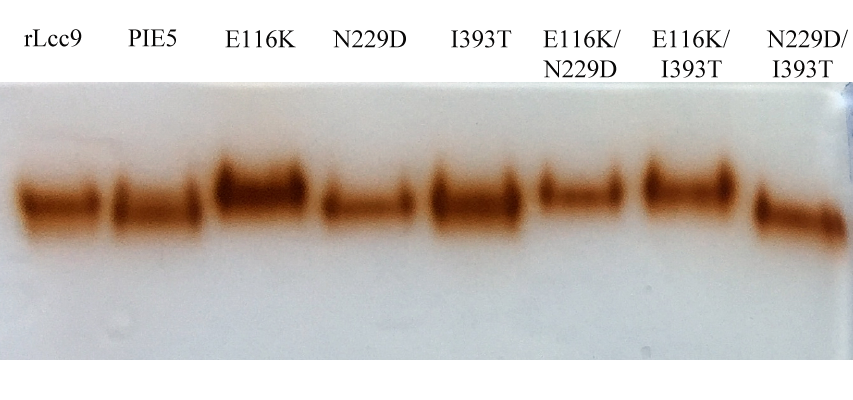

Supplement: Supplementary file 1 — Additional file 1: Table S1. Primers used in this study. Fig. S1. Sequence alignment of Lcc9 and laccase sequences from other basidiomycetes. ▲ indicates the mutated amino acids. Fig. S2. Stereo views of the three types of cupper binding sites in C. cinerea Lcc9 and the mutated sites in variant PIE5. The copper atoms, one type-1 (T1), one type-2 (T2), and, two type-3 (T3a and T3b), are coordinated by the surrounding ten conserved histidines, one conserved cysteine and two water molecules. Protein residues are shown as a link model, the oxygen atoms are shown in red, nitrogen in blue, sulfur in yellow, and carbon in green. The four copper ions and water (Wat1 and Wat2) molecules are represented by blue and red spheres, respectively. a, rLcc9; b, PIE5. Fig. S3. Native PAGE analysis of the purified rLcc9, PIE5, and the specific mutants. Proteins were stained with 1 mM guaiacol in citrate/phosphate buffer (pH 4.0). [file 13568_2019_878_MOESM1_ESM.docx]
